# The Promise of Synthetic Biology for Redesigning Plant Architecture

**DOI:** 10.3390/ijms27114876

**Published:** 2026-05-28

**Authors:** Suruchi Roychoudhry, Gerard D. dos Santos, James P. B. Lloyd

**Affiliations:** 1 School of Biology, University of Leeds, Leeds LS2 9JT, UK; bs22gdds@leeds.ac.uk; 2 ARC Centre of Excellence in Plants for Space, School of Molecular Sciences, The University of Western Australia, Perth 6009, Australia; 3 ARC Centre of Excellence in Plants Energy Biology, School of Molecular Sciences, The University of Western Australia, Perth 6009, Australia

**Keywords:** plant synthetic biology, developmental reprogramming, developmental plasticity, gene editing, gene circuits, programmable plants

## Abstract

Ensuring global food security under accelerating climate change requires transformative approaches to crop improvement that extend beyond the limits of traditional breeding and gene editing. While domestication and modern agriculture have delivered substantial gains in productivity, these advances often came at the cost of genetic diversity, stress resilience, and developmental plasticity. Plants, however, inherently exhibit remarkable flexibility in their morphology and development, as evidenced by the vast diversity of organ shapes, cell types, and adaptive responses that have evolved across lineages. This natural design space provides a foundation for reimagining plant architecture using synthetic biology. Recent advances in plant synthetic biology, including programmable transcription factors, CRISPR-based regulatory systems, synthetic gene circuits, orthogonal signalling pathways, and plant artificial chromosomes, now enable precise, modular, and environmentally responsive manipulation of developmental processes. These tools allow researchers to rewire hormone pathways, tune quantitative gene expression, integrate multiple environmental signals, and create novel regulatory modules that operate independently of endogenous networks. Beyond understanding plant development, these capabilities open avenues for engineering crops with dynamic architectures, enhanced plasticity, and improved resilience to complex and fluctuating stresses. In this review, we synthesise insights from natural diversity, developmental biology, and synthetic regulatory engineering to outline how plant architecture can be rationally redesigned. We argue that integrating synthetic biology with modern breeding and modelling frameworks will be essential for generating the next generation of programmable crops; i.e., varieties capable of sustaining productivity and stability in an era of unprecedented environmental and geopolitical changes.

## 1. Introduction

Ensuring sufficient food production for a growing global population remains one of the most pressing challenges of the 21st century. With the world population projected to peak in the 2080s and the availability of arable land continuing to decline, agricultural systems must deliver significantly higher yields and resource-use efficiency. These pressures are intensified by climate change, which is expected to increase the frequency and severity of extreme weather events—including heatwaves, droughts, and flooding—and thereby threaten global food security. Although technological innovations such as the Haber–Bosch process and the Green Revolution dramatically increased productivity in the 20th century, gains in yield have plateaued in many regions, while others have experienced stagnation or even declines in crop performance [1] in the 21st century. As climate impacts intensify and geopolitical instability increasingly intersects with food insecurity, transformative approaches to crop improvement will be required to sustain global food systems.

One promising avenue lies in synthetic biology, broadly defined as the application of engineering principles to construct novel biological components or redesign existing genetic systems. Synthetic biology has long been proposed as a means to address global challenges [2,3], yet its application in plant biology has lagged due to technical, ethical, and regulatory constraints [2,4]. Nonetheless, advances in genome engineering, gene circuit design, and developmental biology now suggest that it may be possible to reprogram how plants perceive environmental cues and execute developmental decisions. Such capabilities could enable the creation of crops that dynamically adjust their architecture, stress responses, or resource allocation strategies in ways not accessible through conventional breeding.

**Figure 1:**
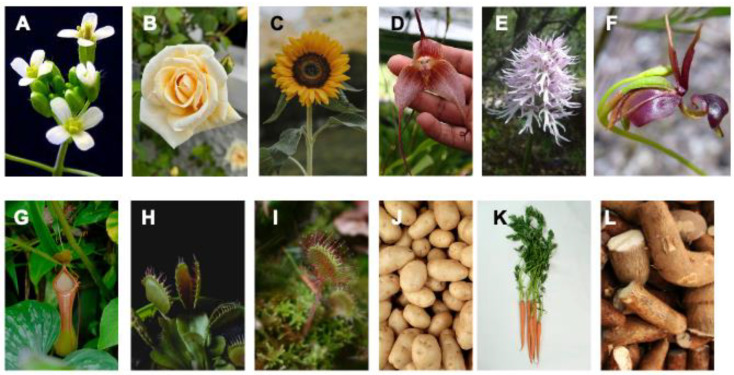
Representative examples of the diversity in plant form, including in organs such as flowers (**A**–**F**), more specialised organs such as traps (**G**–**I**) and root structures (**J**–**L**). (**A**) Flowers of *Arabidopsis thaliana*. (**B**) A flower of a rose, *Rosa chinensis*. (**C**) A flower of a sunflower, *Helianthus annuus*. (**D**) A flower of the monkey face orchid, *Dracula simia*. (**E**) A flower of the naked man orchid, *Orchis italica*. (**F**) A flower of the flying duck orchid, *Caleana major*. (**G**) The cup-trap of the common swamp pitcher-plant *Nepenthes mirabilis*. (**H**) The traps of a Venus Fly Trap, *Dionaea muscipula*. (**I**). A trap of the common sundew plant, *Drosera rotundifolia*. (**J**) The tuber of potato, *Solanum tuberosum*. (**K**) The taproot of carrot, *Daucus carota*. (**L**) The storage root of cassava, *Manihot esculenta*.

Plants inherently possess remarkable developmental plasticity, enabling a single genotype to generate diverse phenotypes depending on environmental conditions. Across species, this plasticity gives rise to an extraordinary range of plant forms (Figure 1), demonstrating the evolutionary flexibility of plant developmental programmes. Domestication has harnessed this variability through selection for traits such as reduced branching, altered flowering time, or compact growth forms [5]. Modern gene editing tools have accelerated the introduction of desirable phenotypes into crops [6]; however, domestication has also narrowed genetic diversity, often at the cost of stress resilience and plastic responses to the environment [7,8].

**Figure 2:**
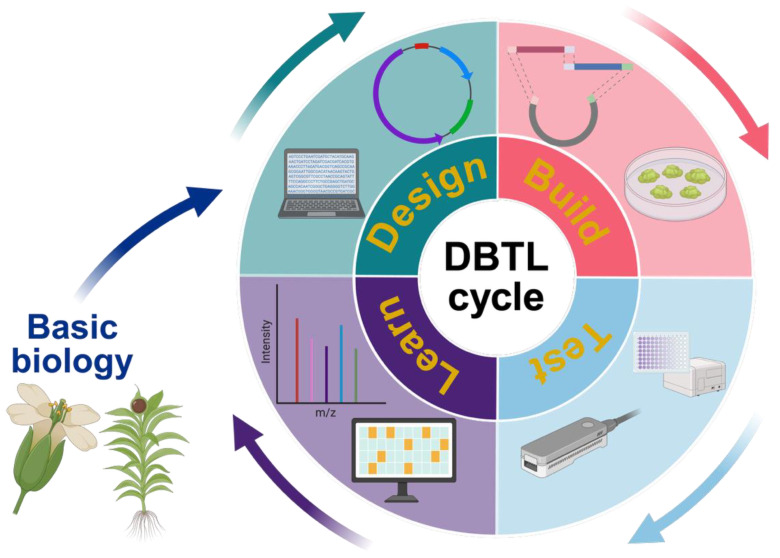
Schematic overview of the Design–Build–Test–Learn (DBTL) cycle of plant synthetic biology. The stages consist of (1) Design: Utilising findings from plant developmental biology combined with computational software to model and design genetic circuits, metabolic pathways, or plants tailored for specific functions (e.g., metabolic production of compounds). (2) Build: Executing the design through DNA synthesis, assembly, and cloning, or CRISPR-based genome engineering, to construct or modify the plant’s genome. (3) Test: Characterising the engineered plants using high-throughput screening and analytical techniques (e.g., omics, HPLC, biosensors) to measure performance, such as growth or yield. (4) Learn: analysing the test data using machine learning and statistical models to understand the system and provide insights for redesigning, creating a feedback loop to guide the next iteration. Figure created with Biorender.com. Lloyd, J. P. B. (2026), https://BioRender.com/.

In this review, we first examine the breadth of natural diversity and plasticity in plant form, with particular attention to how these features have been shaped through domestication and breeding. We then explore how synthetic biology can be used to dissect and re-engineer the developmental processes that underlie plant architecture through a typical ‘Design–Build–Test–Learn (DBTL)’ cycle (Figure 2). Finally, we consider the opportunities and challenges associated with applying engineered developmental programmes to enhance crop performance and resilience. Together, these perspectives highlight the emerging potential of synthetic biology to transform how we design plant structure for the agricultural systems of the future.

## 2. Modern Plant Breeding Has Resulted in the Loss of Resilience Traits

Modern plant breeding has played a central role in expanding agricultural productivity, yet it has also contributed to the unintentional erosion of traits underlying environmental responsiveness and stress tolerance [7,8]. In particular, genetic erosion refers to the loss of genetic diversity, ranging from the loss of individual genes or alleles to the disappearance of entire varieties and crop species. This is driven primarily by the replacement of traditional landraces with modern high-yielding cultivars, natural disasters, and extensive habitat destruction affecting wild species. For example, in small, fragmented populations, genetic erosion limits adaptation by reducing adaptive genetic variation through genetic drift and lowering fitness through inbreeding depression, thereby decreasing tolerance to environmental stress [9].

Domestication and subsequent breeding have also imposed strong genetic bottlenecks on many crops, narrowing allelic diversity and eliminating variants associated with resilience to drought, salinity, temperature extremes, and fluctuating environments. For example, the reduction in genetic diversity induced by bottle-neck domestication has already been well documented in common bean [10]. While wild progenitors and landraces evolved under diverse and challenging ecological conditions, modern breeding prioritised uniformity and high-yield in managed, high-input agricultural environments, substantially reducing the potential of elite cultivars to acclimate to new stressful environments.

One major consequence of this genetic narrowing is the loss of phenotypic plasticity, i.e., the ability of plants to modify their growth and development in response to environmental cues. Plasticity is fundamental to resilience, enabling plants to maintain fitness across heterogeneous or unpredictable conditions. However, breeding for stability and uniformity has frequently reduced genotype-by-environment (G × E) interactions. Studies in crops such as maize and rice indicate that artificial selection has constrained plastic responses in key traits, including flowering time, shoot architecture, and root system dynamics [11,12]. Domesticated varieties of crops outperform wild relatives under favourable, high-input conditions, but this advantage is lost under the increasing frequency and magnitude of extreme weather events such as prolonged drought [13]. This is because wild relatives exhibit greater phenotypic homeostasis, maintaining more stable productivity across environments [8]. Domesticated crop varieties have been described as the “master of some,” due to their high performance in ideal conditions but poor performance in stressful ones [8]. This reduction in phenotypic plasticity diminishes the capacity of modern cultivars to cope with climate variability. For instance, the development of high-yielding semi-dwarf rice varieties during the early domestication of rice favoured responsiveness to nitrogen fertiliser but inadvertently eliminated adaptive flooding responses present in many traditional landraces [14]. These modern varieties generally lack the elongation capacity required to survive prolonged submergence. Efforts to restore this resilience through the introgression of the ***Submergence** 1* (*Sub1*) locus into elite rice cultivars have successfully improved submergence tolerance without sacrificing yield potential in flash flood-prone regions such as India, Bangladesh and Southeast Asia, demonstrating the value of traditional germplasm for climate-resilient breeding [15,16,17].

Trade-offs between yield and stress resilience extend beyond plasticity. Selection for high productivity has often resulted in the loss of alleles conferring tolerance to abiotic stresses. For example, salt tolerance in ancestral tomato species is mediated by *SOLANUM LYCOPERSICUM* HIGH-AFFINITY K^+^ (POTASSIUM) TRANSPORTER 20 (SlHAK20), a Na^+^/K^+^ transporter that maintains ionic homeostasis under high salinity. Many modern tomato cultivars carry loss-of-function mutations in this gene, rendering them more salt-sensitive [18]. A similar pattern is observed in wheat evolution: HIGH AFFINITY POTASSIUM TRANSPORTER1;5-A (TmHKT1;5-A), a plasma membrane Na^+^ transporter that enhances salt tolerance in *Triticum monococcum* (Einkorn), is absent from both durum and bread wheat genomes [19]. These cases illustrate how domestication and selection for desirable agronomic traits can inadvertently eliminate regulators essential for stress tolerance [20].

Other breeding decisions have revealed metabolic trade-offs. Enhancing disease resistance or abiotic stress tolerance through selection or genetic engineering sometimes imposes penalties on growth, seed production, or metabolic balance. For instance, manipulations of amino acid transporters have improved stress tolerance in several species but frequently reduced seed weight or altered metabolite profiles, reflecting the energetic and physiological costs of defence [21]. Such trade-offs underscore the difficulty of simultaneously optimising growth and survival traits within traditional breeding frameworks.

To address these limitations, researchers are increasingly exploring rewilding and *de novo* domestication strategies. Rewilding reintroduces lost resilience traits from wild relatives into elite cultivars, whereas *de novo* domestication uses genome editing to develop new crops directly from wild species that naturally exhibit high stress tolerance and adaptability [7]. These approaches aim to restore environmental responsiveness without compromising yield, leveraging modern tools such as CRISPR-based gene editing, multi-omics selection pipelines, and high-throughput phenotyping. Despite their promise, rewilding and *de novo* domestication face several limitations. Rewilding is constrained by the genomic incompatibilities and linkage drag that can accompany introgression from wild relatives, often reintroducing undesirable traits alongside beneficial alleles. Many resilience traits in wild species are also polygenic and environmentally sensitive, making them difficult to transfer in a predictable manner. Meanwhile *de novo* domestication, accelerated by genome editing, remains limited by incomplete knowledge of the genetic architecture underlying domestication traits and by the long generation times, complex genomes, or recalcitrant transformation systems of many wild species. Both approaches are further restricted by regulatory hurdles, the availability of high-quality reference (pan)genomes, and the need for extensive field testing to ensure agronomic viability. Together, these constraints highlight the need for complementary strategies to engineer resilience traits that are difficult to recover through conventional or wild germplasm-based breeding. In this light, synthetic biology offers the possibility of engineering entirely new regulatory mechanisms, stress-response circuits, or architectural programmes that were never present—or were lost—during domestication. By constructing synthetic sensors, synthetic promoters, or rewired developmental pathways, it may become possible not only to restore lost plasticity, but to design *programmable* resilience traits that allow crops to respond dynamically to environmental fluctuations. Further, synthetic gene circuits can be designed to control plant traits with far greater precision than is currently possible. Reversible circuits may be suited to traits that help plants to withstand temporary environmental stresses, whereas irreversible activation is advantageous for traits like high-value metabolite production once sufficient biomass has accumulated. Although plants currently lack robust tools to dictate when and where genes are expressed, often leading to pleiotropic side effects, rational circuit design offers a path to overcome these limitations [4]. Engineered circuits could enable traits such as nutrient-responsive root architecture or timed induction of protective pathways aligned with predicted weather events, especially as more advanced synthetic sensors become available. In this way, synthetic biology complements and extends traditional breeding, providing a toolkit for reinstating adaptability and creating next-generation crops capable of thriving in increasingly unpredictable climates.

## 3. Diversity of Plant Organ Morphology as a Blueprint for Bioengineering at Organ and Cellular Levels

Evolution has generated an extraordinary breadth of plant structures, both at the organ and cellular levels. Many plant organs have evolved multiple times independently—leaves and roots, for example, have arisen repeatedly during land plant evolution [22]—demonstrating that similar forms can be reached through distinct evolutionary trajectories. This repeated innovation suggests that developmental programmes are highly adaptable, and that plant form is deeply linked to function.

Plants produce a vast array of organ shapes, from simple to highly elaborated forms (Figure 1). Floral diversity alone spans from the modest flowers of *Arabidopsis thaliana* (Figure 1A) to the multilayered blooms of roses (Figure 1B), the composite flower heads of sunflowers (Figure 1C), and the strikingly patterned orchids such as *Dracula simia* (Figure 1D), *Orchis italica* (Figure 1E), and *Caleana major* (Figure 1F). Other organs exhibit similar diversity: leaf-derived traps in *Nepenthes mirabilis* (Figure 1G), snap traps in *Dionaea muscipula* (Figure 1H), sticky glandular leaves in *Drosera rotundifolia* (Figure 1I), swollen stems of potato tubers (Figure 1J), large taproots such as those of carrot (Figure 1K), and starchy storage roots in cassava (Figure 1L).

This diversity reflects both convergent evolution, where similar structures evolve independently, and divergent evolution, where a common ancestral structure radiates into multiple forms. For example, cup-shaped leaves in carnivorous plants have evolved at least four times independently [23]. Within the bladderwort *Utricularia*, divergent evolution has produced substantial variation in trap size and shape [23]. One of the most striking instances of convergent evolution is the >60 independent origins of C_4_ photosynthesis [24,25], which required coordinated changes in leaf development (Kranz anatomy) and metabolism. Despite the repeated evolution of C_4_ lineages, attempts to engineer C_4_-like traits into C_3_ crops such as rice have so far produced only incremental progress, underscoring the complexity of this transition [26].

The duckweed family provides another example of dramatic natural developmental reprogramming. These aquatic plants descended from terrestrial ancestors related to aroids but underwent severe morphological reduction, losing roots, stems, and complex leaves as they adapted to floating aquatic habitats [27]. Genomic analyses reveal widespread gene loss and streamlining, along with expansions in pathways linked to rapid growth and nutrient uptake—highlighting how evolution can radically reshape plant body plans.

Together, these examples show that plant organs can be extensively remodelled over evolutionary time. Although current biotechnological approaches cannot yet rationally design organs de novo, they suggest a long-term roadmap in which synthetic biology could enable precise changes to organ shape, cell patterning, and tissue organisation, or even the creation of novel synthetic organs. For example, synthetic biology tools have already been used to design ‘organoids’ in human biology. Organoids have been defined as in vitro 3D structures that display important aspects of the anatomy and physiology of their in vivo counterparts and that develop from pluripotent or tissue-specific stem cells through a self-organisation process [28]. It is highly likely that similar engineering biology approaches could be implemented to design in vitro plant organoids capable of specialised functions in the near future.

## 4. Cellular-Level Innovation and Developmental Plasticity

Plant evolution has also produced remarkable diversity in cell types—root layering systems, stomatal guard cells, trichomes, storage parenchyma, and more—each specialised for transport, protection, or metabolic functions. These cell types arose as plants adapted to terrestrial challenges such as desiccation, mechanical load, and nutrient scarcity, driven largely by changes in gene regulation and developmental patterning.

One striking example of cellular plasticity is callus formation. Once thought to represent a mass of “undifferentiated cells,” callus is now understood to be a root-related developmental state, co-opting gene networks associated with lateral root formation [29,30,31,32]. The ability to induce callus in vitro and regenerate entire plants through controlled application of auxin and cytokinin exemplifies the unusually high developmental potency of plant cells. Unlike animal induced pluripotent stem (iPS) cells, which often retain epigenetic barriers that limit their capacity to differentiate into all cell types, plant cells exhibit far greater flexibility in cell fate [33], although not all cells are equally competent. For instance, *Arabidopsis* root explants cannot directly convert to shoots without a callus intermediate unless the DNA methyltransferase *CHROMOMETHYLASE 3* (*CMT3*) is knocked out, enabling direct root-to-shoot transitions [34]. This indicates that chromatin state still constrains fate switching in some contexts.

The terminology surrounding callus has therefore shifted, with “transdifferentiation” or “over-proliferating lateral root primordia” considered more accurate than “dedifferentiation” [35]. Importantly, several studies demonstrate that manipulating developmental regulators can reassign organ identity. In the *topless* (*tpl*) mutant of *Arabidopsis*, root identity replaces the shoot apical meristem at low frequency [36]. Conversely, targeted manipulation of meristem identity genes can convert root meristems into shoot meristems, generating seedlings with duplicated shoots [37]. While these examples have limited current agricultural use, they highlight the tremendous potential of developmental engineering to reshape plant architecture.

Synthetic biology could ultimately harness such mechanisms to induce or stabilise new cell fates, engineer artificial meristems, or control multicellular patterning with high precision.

The above examples showcasing the extraordinary range of plant forms arising through convergent and divergent evolution demonstrate that both organs and cells are highly malleable systems. For bioengineers, this diversity provides a conceptual “design space” of solutions that evolution has already validated. The major barriers to re-engineering plant structure are therefore not inherent biological constraints, **but rather our current lack of predictive understanding of how genetic perturbations influence developmental trajectories**. As synthetic biology tools expand, such as programmable gene circuits, synthetic promoters, spatial patterning modules, and engineered signalling pathways, they offer a means to increasingly harness, emulate, or surpass the natural innovations produced by plant evolution (see Figure 2).

## 5. Developmental Biology for Crop Improvement

Structural traits that enhance resource allocation and stress resilience underpin a plant’s ability to maintain productivity under variable environmental conditions. Optimised plant architecture, including ideal leaf angles, robust stems, and efficient root systems, improves light interception, nutrient acquisition, and resistance to lodging, thus collectively contributing to higher yields. The profound influence of plant architecture on productivity was exemplified during the Green Revolution, when semi-dwarf varieties of rice and wheat dramatically increased global yields. For example, between 1960 and 2000, yields for all developing countries rose 208% for wheat, 109% for rice, 157% for maize, 78% for potatoes, and 36% for cassava. Developing countries in southeast Asia and India were the first countries to show the impact of the GR varieties on rice yields, with China and other Asian regions experiencing stronger yield growth in the subsequent decades [38]. Similar yield trends were observed for wheat and maize in Asia [38]. Reductions in plant height, achieved through manipulation of gibberellic acid (GA) signalling, produced cultivars with reduced lodging and increased tiller number. In wheat, this was largely driven by mutations in the *Reduced height* (*Rht*) gene family, particularly the homoeologues *Rht-B1b* and *Rht-D1b*, which confer GA insensitivity and remain foundational to modern high-yielding cultivars [39]. This success underscores the transformative potential of targeted genetic and synthetic biological interventions in plant development.

Beyond plant stature, a range of architectural traits, including leaf and branching angle, leaf morphology, flowering time, and vascular design, contribute to photosynthetic capacity and the efficient movement of photosynthates from source to sink [40]. C_4_ photosynthesis represents a particularly striking example of developmental and biochemical optimisation working in concert to boost productivity. At the biochemical level, C_4_ plants use a CO_2_-concentrating mechanism in which phosphoenolpyruvate carboxylase (PEPC) initially fixes CO_2_ in mesophyll cells, forming oxaloacetate, which is shuttled to bundle sheath cells for decarboxylation and refixation by Rubisco. Developmentally, this process is enabled by Kranz anatomy, where mesophyll and bundle sheath cells are arranged to facilitate spatial separation of the C_4_ pathway. C_4_ photosynthesis has evolved independently over 65 times, highlighting its strong adaptive value in hot, high-light, and low-CO_2_ environments. Together, the biochemical and anatomical features of C_4_ plants confer superior photosynthetic efficiency as well as improved water and nitrogen use efficiency, contributing to high yield potential in challenging environments. This is indeed a promising area for crop improvement, and work is ongoing to not only enhance the carboxylation capacity of Rubisco, but also enhance the generation of ATP generation and light-harvesting in bundle sheath cells [41,42]. Intriguingly, researchers have also begun to propose the engineering of a simplified rational C_4_ photosynthetic pathway with known C_4_ metabolic components in C_3_ plants using synthetic biology [43]. Alternatively, the development of a recent grafting technique for monocots at the embryonic root–shoot interface has enabled fusion of C_4_ rootstocks with C_3_ shooting architecture, overcoming previous limitations across the vascular cambium and enabling, for instance, the grafting of cereals to rootstocks to confer resistance to soil-borne pathogens [44]. More recent work has yielded promising preliminary results showing an average yield increase of 24.5% in transgenic soybean with engineered photosynthetic capacity [45].

Below ground, root architecture is equally critical for crop resilience and productivity. A well-developed root system improves anchorage and supports efficient uptake of water and nutrients, thereby sustaining photosynthetic activity and reproductive output. Root system plasticity further allows crops to adapt to spatially and temporally heterogeneous soils, promoting yield stability under field conditions. Key traits such as root depth, the degree and density of secondary branching, root growth angle and root hair production directly influence soil exploration and resource acquisition, especially under drought or nutrient limitation. A landmark example is the identification of the *DEEPER ROOTING 1* (*DRO1*) gene in rice, which regulates root growth angle [46]. Introgression of *DRO1* into shallow-rooting cultivars produced lines with deeper roots, superior drought avoidance, and significantly higher yield under water-limited conditions. Further, *ENHANCED GRAVITROPISM1* (*EGT1*) and *ENHANCED GRAVITROPISM2* (*EGT2*) were recently shown to regulate root growth angle in barley and wheat. In maize, the “steep, deep, and cheap” ideotype proposed by Jonathan Lynch further emphasises the value of deep, resource-efficient root systems for optimising nitrogen and water uptake [47].

Other studies have highlighted the roles of hormone signalling networks in regulating root architecture. For example, the disruption of auxin signalling and transport in rice has been shown to reduce crown and lateral rooting density whilst increasing root hair and density and length [48].

Such developmental insights open compelling opportunities for synthetic biology. Engineered gene circuits could, in principle, allow real-time reprogramming of root growth in response to dynamic environmental cues; for example, promoting deeper rooting during drought or nitrate scarcity, while favouring shallower, more proliferative roots under phosphate limitation. This level of environmentally responsive architectural tuning could enable highly adaptive crops capable of maintaining productivity under fluctuating climatic and edaphic conditions.

Advances in rice architecture further illustrate the yield potential of targeted developmental modification. For example, the *TILLER ANGLE CONTROL 1* (*TAC1*) gene regulates tiller angle, and alleles promoting near-vertical tiller growth result in more compact plant forms with improved light interception and increased yield potential [49]. Together, these studies demonstrate how precise manipulation of developmental traits—whether through traditional breeding, genomics-informed approaches, or synthetic biology—can drive substantial gains in crop productivity and resilience.

## 6. Current State of Genetic Intervention in Crops

For millennia, humans have shaped plant phenotypes through selection for traits advantageous for cultivation, nutrition, or harvestability. Traditional domestication relied entirely on observable phenotypes, with no understanding of the underlying genetic variants being selected. As a result, pre-existing genetic diversity within crop populations was gradually enriched for favourable alleles. Modern genome-assisted breeding now enables breeders to track and combine these alleles more efficiently; however, the fundamental principle remains unchanged: existing variation is rearranged, not newly created.

Much of the diversity observed in contemporary crops reflects genomic changes occurring across multiple evolutionary and human-mediated scales. These range from single-nucleotide mutations—sometimes induced by mutagens such as gamma irradiation—to extensive structural changes, including whole-genome duplications. For example, triploidy underlies the development of larger, seedless bananas, while a single premature stop codon in the *GRAIN SIZE 3* (*GS3*) gene in *Oryza sativa* is responsible for long-grain rice varieties [50,51,52]. Although induced mutagenesis has occasionally been used to expand the genetic landscape of breeding material, such approaches rely on random mutation events and provide little control or predictability.

The emergence of gene-editing (GE) technologies, most notably CRISPR-Cas systems, has enabled precise, user-defined mutagenesis. By guiding a Cas nuclease to a specific genomic locus via a single guide RNA (sgRNA), researchers can induce targeted double-strand breaks and exploit endogenous DNA repair pathways to introduce defined mutations. Importantly, gene editing can recreate alleles that might arise naturally but enriches for them at loci of known functional significance, thereby increasing the likelihood of obtaining a desirable phenotype compared to random mutagenesis. For this reason, many regulatory agencies treat gene-edited crops differently from transgenic crops, and in several jurisdictions GE products are exempt from the stringent regulations applied to genetically modified organisms (GMOs). The EU is currently evaluating reforms that may relax restrictions on certain gene-edited crops [53]. Gene editing is now also being applied to modify plant architecture directly. For instance, recent work in the “minor crop” goldenberry leveraged CRISPR to produce compact, farmer-friendly growth habits [6]. Many similar applications are emerging across crop species.

Genetic modification (GM), in contrast, involves the introduction of novel DNA, often from another species, into a plant genome. The introduced sequence, or transgene, is stably integrated via transgenesis. Unlike GE-induced edits, such cross-species DNA movement is rare in nature, though horizontal gene transfer does occur. *Agrobacterium tumefaciens*, for example, naturally transfers DNA into plant genomes during infection. Remarkably, modern sweet potato cultivars carry *Agrobacterium*-derived T-DNA insertions, inherited from an ancestral infection event [54,55]. Despite containing naturally occurring foreign DNA, sweet potatoes are not regulated as GM crops, illustrating inconsistencies in regulatory logic.

Public perception of GM crops remains mixed, and regulatory restrictions persist in many regions. Despite this, GM agriculture remains globally significant: in 2023, GM crops were cultivated on approximately 13% of all arable land, with the overall global footprint continuing to expand rather than decline [56]. This suggests that developing a transgenic crop remains a viable route for agricultural deployment, particularly in areas facing acute food insecurity. Historical resistance to GM technologies, such as protests and destruction of GM field trials in the UK, or the “great petunia carnage of 2017,” when inadvertently released GM petunias were culled across the EU [57,58], illustrates societal tensions, though such events may become less common as climate pressures intensify.

The earliest generations of GM crops relied on constitutive expression of “always-on” transgenes to confer herbicide tolerance or insect resistance [4,59]. In many cases, these imposed little metabolic burden, enabling widespread adoption. However, some transgenes cause undesirable pleiotropic effects when expressed continuously, such as pathogen-defence genes that impair growth [60,61] or pathways engineered to overproduce oils in leaves [62]. Synthetic biology offers solutions by enabling fine-grained spatial and temporal regulation of transgene expression, minimising negative side effects while retaining beneficial traits [2]. In parallel, transgenic trait engineering has expanded from single-gene approaches to multigene trait stacks, as demonstrated by Golden Rice [63,64,65] or omega-3 fatty-acid-producing canola and tomato varieties [66,67,68].

Given the diversity of modern genetic technologies, several researchers have proposed that regulation should shift from focusing on *how* a trait is introduced (random mutagenesis, gene editing, transgenesis) to evaluating the *trait itself* and its environmental impact [2,69]. If public perception evolves and regulators adopt trait-centric frameworks, it is plausible that more advanced GM and synthetic-biology-derived crops will reach the field. Such progress could create a positive feedback loop: successful deployment of advanced engineered traits may drive increasing societal acceptance, enabling transformative agricultural innovations at scale.

## 7. Recent Advances in Plant Synthetic Biology

Plant synthetic biology has expanded rapidly in recent years, enabling precise and orthogonal manipulation of plant gene expression, metabolism, and development (See Table 1). The field now offers a diverse toolkit for engineering biomolecules, pathways, and regulatory networks, providing unprecedented opportunities to reshape plant form and function.

**Figure 3:**
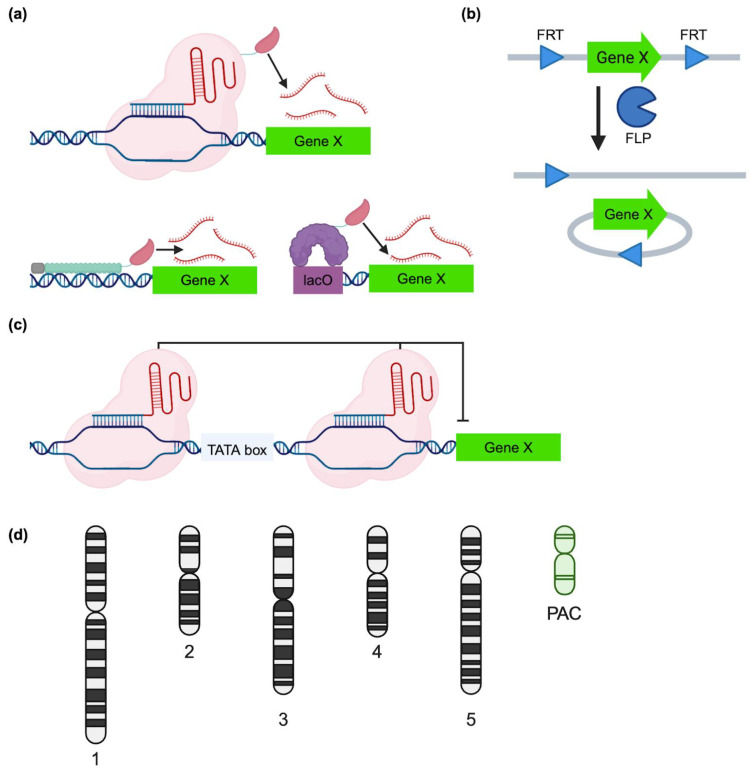
Molecular tools from synthetic biology that can be used to alter plant development. (**a**) Synthetic transcription factors can be made from CRISPR-dCas9, TALE or bacterial transcription factor proteins fused to a transactivation domain to induce transcriptional activity. (**b**) Gene activity can be modified by recombinase (e.g., FLP) to remove genic components, such as the coding sequencing (CDS) of the gene, and cause permanent repression (NOT gate). (**c**) CRISPR interference can be created via CRISPR-dCas9 in critical regions of a plant promoter, such as surrounding a TATA box to repress the target gene. Two dCas9 that can bind to the same promoter via different sgRNAs form a NOR gate. (**d**). The introduction of a plant artificial chromosome (PAC) in the nucleus alongside the native chromosomes of the plant. Figure created with Biorender.com. Lloyd, J. P. B. (2026), https://BioRender.com/.

A major area of innovation has been the development of **synthetic transcription factors (synTFs)**. SynTFs can be rationally designed to bind and regulate user-defined genomic targets (Figure 3a). One widely adopted approach uses nuclease-dead Cas9 (dCas9) fused to transcriptional effector domains such as VP64, with a sgRNA directing dCas9 to the desired locus [70] (Figure 3a). Depending on the effector fused to dCas9, synTFs can activate or repress transcription. Notably, Khakhar et al. engineered **hormone-activated Cas9-based repressors (HACRs)**, in which dCas9 is combined with a hormone-responsive degron and a TOPLESS-derived repressor domain [71]. In the presence of specific phytohormones, HACRs are degraded, alleviating repression of the target gene. Application of an auxin-degradable HACR targeting *PIN1* resulted in decreased shoot branching and phyllotactic noise, validating previously published models. Collectively, these studies demonstrate how synthetic regulators can reprogram developmental pathways in predictable ways. [71].

In addition to CRISPR-based synTFs, **TALE (transcription activator-like effector) proteins** provide a protein-engineered alternative (Figure 3a). Their modular DNA-binding repeats can be assembled to recognise virtually any sequence, and when fused to activation domains, they function as designer transcription factors [72,73]. An older, yet still powerful, strategy fuses eukaryotic activation domains to bacterial DNA-binding proteins such as LacI, enabling orthogonal control when paired with synthetic operator sites (lacO) inserted into plant genomes (Figure 3a). This system has supported hormone-responsive switches [74] and the construction of synthetic regulatory circuits [75]. Epigenome-editing tools, such as targeted DNA methylation modifiers, have further expanded the capacity to tune endogenous loci without altering their sequence [33].

**Site-specific recombinases**, including CAUSES RECOMBINATION (CRE), FLIPPASE (FLP), and others derived from bacteriophage or yeast, offer another class of synthetic biology tools for genome engineering, lineage tracing, and regulated gene expression (Figure 3b). For example, FLP recombinase recognises 35 bp *FRT* sites and excises sequences flanked in the same orientation, leaving a stable recombination scar [76,77]. These systems enable irreversible, user-defined modifications such as gene removal or promoter swapping.

CRISPR-based regulatory approaches extend beyond activation and repression. **CRISPR interference (CRISPRi)** uses dCas9 binding to sterically hinder transcription initiation (Figure 3c). Khan et al. (2025) [78] showed that by inserting high-affinity sgRNA-binding sequences near the TATA box of a modified 35S promoter, transcription could be repressed by up to 95% (Figure 3c). They further engineered sgRNA expression systems that respond to RNA polymerase II promoters via CSY4-mediated processing, enabling inducible repression in response to environmental cues such as heat shock.

These components have facilitated the emergence of **synthetic gene circuits**, which function as programmable regulatory networks analogous to Boolean logic gates. Circuits enable plants to process environmental inputs, such as stress signals, and convert them into defined genetic outputs, including activation or repression of developmentally relevant regulators [4,79]. For example, CRISPRi systems with two sgRNA sites can implement a NOR gate [78], recombinases can build irreversible NOT gates [76], and bacterial transcription factors can form YES/BUTTON gates or buffering modules [75]. Multiple platforms now exist for constructing such circuits using CRISPRi [78,80], recombinases [76,81], or orthogonal transcription factors [75].

Beyond regulatory tools, efforts are underway to expand the infrastructure for synthetic genetics in plants. **Plant artificial chromosomes (PACs)** represent a promising frontier (Figure 3d). PACs are broadly defined as synthetic chromosomes inherited through mitosis and meiosis and could be built via top-down truncation of native chromosomes or via *de novo*, bottom-up assembly [82,83]. Although bottom-up PAC synthesis remains technically challenging, it offers the advantage of avoiding disruption of essential endogenous genes. PACs could provide stable genomic landing pads for large multigene trait stacks, insulated from position effects and linkage drag. Insights from human artificial chromosome (HAC) engineering, including synthetic centromeres assembled from lacO arrays recruiting LacI-centromere fusion proteins [84,85], point toward feasible strategies for PAC development. A top-down approach to create a PAC is the fission of natural chromosomes by the insertion of a neocentromere, which has been achieved in maize [86]. Key questions, such as the minimum chromosomal size required for faithful segregation, remain active areas of research [83]. Their use could overcome breeding limitations related to linkage disequilibrium by moving key genes for breeding programmes onto the PAC.

Related genome-scale efforts include genome minimisation and rewriting projects, such as the SynMoss initiative in *Physcomitrium patens*, which leverages its high efficiency of homologous recombination [87,88,89]. These projects aim to define essential elements of plant genomes, including non-coding regulatory regions, and may ultimately facilitate chassis organisms optimised for synthetic biology.

Additional synthetic biology advances have created tools that, while not always directly developmental, significantly expand engineering capabilities. Examples include orthogonal peroxisomal import systems enabling the design of synthetic organelles in *Physcomitrium* [90]; autonomous bioluminescent plants engineered using fungal metabolic pathways [91,92,93,94], now used as biosensors for viral infection [95]; and polycistronic reporters derived from engineered betalain pathways [96]. Rewiring ligand specificity in ABA receptors has yielded engineered sensors responsive to novel agrochemicals [97,98], and the recent creation of orthogonal ligand-induced ABA receptor–co-receptor pairs further expands possibilities for environmentally triggered developmental control [99].

Together, these tools underscore the quickly expanding potential of plant synthetic biology. Some have already been applied to reprogram development, others are accelerating discovery, and many more will undoubtedly enable currently unimaginable innovations. In this regard, a key emerging need is dynamic control over when and where genes are expressed. Gene editing and traditional breeding, while powerful, often produce static changes and may be limited by pleiotropic effects. Synthetic biology, with its orthogonal components and inducible systems, offers a path to re-establish programmable plasticity with fewer trade-offs.

Synthetic biology uniquely allows introduction of genetic modules that function orthogonally—that is, independently of native plant pathways. Using bacterial, fungal, or animal components that interact strongly with one another but minimally with plant machinery allows the design of predictable, insulated gene regulatory programmes that can reshape development without interfering with endogenous processes [4].

Although GM crops remain controversial in many regions, they have a long record of safety, and global challenges may shift public perception. As the climate crisis intensifies and food insecurity worsens, pressure will likely grow to deploy advanced engineered traits. Historically, societal resistance—including, for example, the destruction of GM field trials or incidents like the EU’s 2017 removal of inadvertently disseminated GM petunias—has hindered progress, but this may change under mounting environmental and economic pressures. Given the scale of the challenges ahead, investment in next-generation GM and synthetic biology-enabled crops may be essential for enabling a new, sustainability-focused Green Revolution.

## 8. Synthetic Biology to Reshape Natural Plant Structure

Plants exhibit extraordinary morphological diversity, reflecting the inherent flexibility of developmental programmes that generate plant form (Figure 1). This diversity highlights the potential to restructure plant architecture far beyond the variation accessible through traditional breeding or genome editing alone. While selective breeding and targeted editing have successfully modified traits such as height, tiller number, and flowering time, these approaches rely primarily on existing genetic variation and operate within the constraints of native developmental networks. In contrast, synthetic biology enables the rational design of programmable, orthogonal regulatory systems capable of reshaping plant form with greater precision, scalability, and predictability.

A central strength of synthetic biology lies in its capacity to build novel gene regulatory circuits, rewire endogenous signalling pathways, and introduce orthogonal modules that operate independently of native plant networks. These tools allow direct control over architectural traits including shoot branching, organ orientation, internode elongation, and root system configuration. Notable advances have come from engineering circuits that modulate auxin signalling, a master regulator of plant morphogenesis. Synthetic promoters, tunable transcription factors, and engineered auxin-responsive elements enable spatially restricted, inducible, or graded gene expression patterns, providing quantitative control that surpasses traditional genetic modification [71,75]. Such systems have been employed to alter shoot branching dynamics, modify root branching density, and modulate tropic responses with high precision.

Genome editing technologies further expand this design space. CRISPR-based transcriptional regulators, base editors, and prime editors allow fine-scale modulation of endogenous gene expression without introducing exogenous coding sequences. These tools have been deployed to modify traits such as leaf inclination, internode length, and root growth angle, each governed by complex regulatory networks [100]. Multiplexed editing strategies enable simultaneous modification of multiple loci within a developmental module, offering opportunities to generate novel ideotypes optimised for specific environments or farming systems. However, it is important to note that these approaches are not without their challenges. CRISPR/Cas9 has several limitations in crop improvement. Editing efficiency and precision can vary by species and genomic locus, and off-target mutations may still occur, especially in large, repetitive plant genomes. Many important agronomic traits (such as yield or stress tolerance) are polygenic, making them difficult to improve by editing single genes. In addition, delivering CRISPR components into some crops and regenerating edited plants remains technically challenging, and regulatory uncertainty in some countries can slow field deployment and commercialisation [101].

Beyond rewiring endogenous pathways, synthetic biology facilitates the introduction of orthogonal signalling systems—engineered receptors, synthetic peptide ligands, and modular sensor–actuator modules that respond to inputs not normally perceived by plants. These systems enable plants to detect and respond to exogenous chemicals, specific wavelengths of light, or non-native environmental cues [102,103,104]. When coupled to downstream developmental regulators, such circuits allow external and reversible control of plant architecture, opening the door to highly adaptive crop varieties.

A rapidly emerging frontier is the engineering of developmental plasticity. Plants naturally modify their architecture in response to environmental stimuli such as nutrient availability, light, gravity, waterlogging, and mechanical strain. However, domestication has often reduced this plasticity (see above). Breeding programmes focused on stability can inadvertently constrain plasticity further, yielding crops optimised only for narrow environmental conditions, rather than the extreme weather events such as flash floods, intense heatwaves and prolonged drought characteristic of global warming and climate change.

Root system architecture exemplifies the complexity of plastic responses. Nutrients vary dramatically in mobility—water and nitrate are relatively mobile, whereas phosphorus and potassium are spatially restricted—requiring distinct foraging strategies [105]. Unexpectedly, the model plant Arabidopsis exhibits maladaptive root architectural changes that reduce foraging efficiency under phosphorus limitation [106]. Combined nutrient stresses amplify these challenges. Synthetic gene circuits capable of integrating multiple environmental signals through Boolean or analogue logic could enable context-dependent developmental responses unattainable through breeding or static genome edits alone.

Enhancing plasticity is particularly valuable in low-input agricultural systems, where resource heterogeneity is high and external fertilisers or irrigation are limited [107]. Such innovations would therefore disproportionately benefit smallholder farmers and contribute to global food security under increasingly variable climates.

Although the genetic basis of plasticity remains only partially understood and is distinct from the architecture of many other phenotypic traits [108], synthetic biology provides a way to bypass these limitations. Orthogonal pathways can interface with well-characterised developmental regulators, converting fixed genetic programmes into flexible, environment-responsive networks. This approach dramatically expands the design space for plant architecture beyond what natural variation or breeding can achieve. Early demonstrations of this principle are emerging; for example, varying the number of bacterial transcription factor binding sites in a synthetic promoter driving the gain-of-function *solitary root-1* (*slr-1*) allele enabled finely tuned control of lateral root density in *Arabidopsis* [75]. Likewise, the use of auxin responsive synTFs called HACRs have successfully been used to control the number of shoot branches on a plant, in a predictable manner [71]. The targeted expression of hormone biosynthesis genes using synthetic transcription factors has already been used to modify plant height in tomato [109]. The use of a synthetic ABA-responsive promoter to drive *Zea Mays DEEPER ROOTING 1* (*ZmDRO1*) expression to force roots to grow more downwards in drought conditions and encourage water seeking structure [110]. Modification of the natural plant transcription factor in the cytokinin pathway to increase its activity as a transcriptional activator or repressor, decreased or increased the number of lateral roots, respectively [111]. Taken together, these studies indicate that rational design of circuits and synTFs are already effective at altering plant structure in a predictable manner.

Mathematical modelling is further enriching our understanding of how complex 3D shapes emerge and may serve as a guide for synthetic design. Modelling has shed light on how leaf and petal morphologies arise from differential growth patterns [9]. Studies integrating modelling with experiments in the carnivorous plant *Utricularia gibba* revealed that altering the adaxial domain of gene expression is sufficient to switch development between flat leaves and complex trap structures, driven by growth along two interacting polarity fields [112]. Combining such predictive frameworks with synthetic regulatory tools could enable deliberate, bottom-up engineering of plant shapes.

Evolutionary studies of plant morphology provide a powerful framework for defining the traits that synthetic biology can exploit. Across multiple contexts, major morphological innovations have arisen not through the invention of new genes, but through the regulatory redeployment of existing ones. For example, the diversity of floral organs is largely explained by combinatorial and spatial regulation of a small set of MCM1/AGAMOUS/DEFICIENS/SRF (MADS)-box transcription factors [113], while repeated evolutionary transitions between simple and compound leaves result from subtle changes in the timing and domain of *KNOTTED1-LIKE HOMEOBOX* (*KNOX*) gene expression [114]. Similarly, dramatic variation in plant architecture during both natural evolution and crop domestication can often be traced to quantitative modulation of key regulatory hubs such as the *TEOSINTE BRANCHED 1* (*TB1*) or *SQUAMOSA PROMOTER BINDING-PROTEIN LIKE* (*SPL*) transcription factors [115,116]. Even complex traits like C_4_ photosynthesis or pigmentation patterns of fruits and petals have evolved through stepwise regulatory rewiring and pattern-forming networks built from pre-existing components [117,118]. Together, these cases suggest that plant morphology is highly evolvable via incremental, modular changes in gene regulatory networks, offering clear design principles viz. combinatorial control, quantitative tuning, and exploration of intermediate states, for rational engineering of new plant forms.

Collectively, these advances highlight the transformative potential of synthetic biology to reshape plant structure. By integrating programmable gene circuits, precision editing, orthogonal signalling pathways, artificial chromosomes, and computational modelling, researchers can now rationally design plant architectures optimised for yield, resilience, sustainability, or entirely novel functions. In this review, we argue that synthetic biology will be central both to advancing basic developmental biology and to engineering crop varieties with enhanced plasticity, customised forms, and new agronomic traits. As global agriculture faces intensifying challenges, these capabilities may become indispensable for future crop improvement.

## 9. Limitations and Risks

Genetic engineering in plants has a long history; however, the rational design of complex synthetic gene circuits and the deployment of synthetic transcription factors (synTFs) are comparatively recent developments. As a result, substantial challenges remain. A fundamental limitation is that not all plant species are readily transformable, and even among those that are, transformation efficiency and regeneration time vary widely. Some important crops, such as sorghum, remain particularly recalcitrant to transformation [119], whereas others, including potato and tomato, are more routinely amenable to existing protocols. Nonetheless, “routine” transformation should not be conflated with simplicity or speed, as these protocols are often technically demanding and regeneration can still be time-consuming.

Unlike *Arabidopsis thaliana*, the principal model flowering plant, most species cannot be transformed using the floral dip method [120], substantially increasing the technical barriers associated with transgene introduction. In addition, many crops have long generation times, with woody perennials representing an extreme case. Although speed breeding approaches can partially mitigate this constraint in some species [121], the translation of tools developed in *A. thaliana* to crop plants remains slow and uncertain [4]. Even within *A. thaliana* itself, the design–build–test–learn cycle (Figure 2) is considerably longer than in microbial systems, where the majority of synthetic biology advances have historically been made [4]. Further, unintended consequences from counter-productive outcomes cannot be ignored. For example, synthetic genes designed to increase tillering and grain size often led to weaker root systems or branches that produced low-quality seeds, ultimately resulting in lower total viable yields [122]. Another example of the limitations of synthetic biology approaches is in biofortification. Scientists attempting to biofortify crops with essential micronutrients (like Vitamin B1) have faced major metabolic roadblocks. In higher plants, the enzyme responsible for thiamine synthesis (THI1) operates via a “suicide” catalytic mechanism—meaning it is irreversibly destroyed after functioning only once. Synthetic biology efforts to overexpress or mutate this enzyme to bypass the suicide mechanism in crops like rice and tomatoes frequently resulted in the transgene being silenced or causing severe metabolic imbalances in the plant [123].

Beyond the challenges of tool translation (Figure 3), perhaps the greatest difficulty, and risk, lies in the rational engineering of plant form itself. Evolution has repeatedly reshaped plant architecture (Figure 1), demonstrating that dramatic morphological change is physically and biologically possible. However, it may be overly optimistic to assume that humans can presently engineer such transformations in a fully rational manner; for example, to generate designer flowers or entirely novel organs. The Nobel Prize winner François Jacob stated “natural selection does not work as an engineer works. It works like a tinkerer” [124], in a stark reminder that we are trying to rationally design the irrationally designed [4]. Our understanding of how complex three-dimensional structures emerge from gene regulatory networks remains incomplete. Nevertheless, increasing efforts are being directed toward this problem, including the application of mathematical and computational modelling to plant developmental biology [112,125].

**Table 1:** Summary of synthetic biology applications in plant architectural engineering.

Architectural Trait	Evolutionary/ Developmental Basis	Engineering Target(s)	Synthetic Biology Strategy	Outcome/Aim	Key References
Shoot branching	Quantitative auxin- mediated apical dominance (TB1/SPL hubs)	*PIN1*, auxin response genes	Hormone-responsive synTFs (HACRs)	Predictable tuning of branch number	[4,71]
Root branching density	Plastic lateral root development via auxin–cytokinin balance	*IAA14/slr*, auxin downstream targets	Synthetic promoters with bacterial TF binding sites	Graded and spatially controlled lateral root formation	[75]
Root growth angle	Gravitropic setpoint control (*DRO 1*, *EGT* pathways)	*DRO 1*	Stress-inducible synthetic promoters (ABA-responsive)	Deeper rooting under drought conditions	[110]
Leaf and organ size	Quantitative growth control via hormone signalling	Hormone biosynthesis or response genes	Targeted synTF-driven expression	Tunable control of plant height and organ size	[71,109]
Tiller angle/canopy architecture	Domestication of rice architecture (TAC1 module)	*TAC1* and regulators	Genome editing + synthetic regulatory tuning	Compact ideotypes for high-density planting	[47,100]
Root system patterning	Modular GRN logic integrating multiple inputs	Multiple developmental regulators	CRISPRi-based synthetic circuits	Context-dependent architectural responses	[78]
Organ patterning (conceptual)	Combinatorial TF networks (ABC model, KNOX)	MADS-box, KNOX modules	Proposed orthogonal combinatorial circuits	Exploration of novel organ forms	[126,127]
Complex leaf morphology	Polarity-field regulation of growth	Adaxial–abaxial regulators	Modelling-guided rewiring	Switching between planar and complex organ forms	[112]

We suggest that closer collaboration between the plant synthetic biology and developmental biology communities will be essential to address these challenges and advance the rational redesign of plant structures. Importantly, even unsuccessful attempts at rational design are likely to be informative, yielding new insights into gene regulation, developmental processes, or both (Figure 2).

Finally, even if major technical breakthroughs are achieved, the adoption of synthetically engineered crops by farmers and the general public remains uncertain, given the long-standing mistrust of genetically modified organisms. That said, at least 13% of global farmland is currently planted with GM crops, and this proportion continues to increase [56], suggesting a growing acceptance that may extend to new varieties enabled by synthetic biology. Regulatory frameworks that focus on the traits of a crop rather than the specific technologies used to produce them may provide a more pragmatic and scientifically grounded path forward [69].

## 10. Summary and Outlook

The rapid expansion of plant synthetic biology is transforming our ability to understand and manipulate plant development. From precision genome editing and programmable transcriptional regulators to orthogonal signalling systems and artificial chromosomes, these tools collectively allow researchers to rewire plant architecture beyond the constraints imposed by natural variation. Such approaches complement, rather than replace, traditional breeding and genome editing by enabling dynamic, modular, and environmentally responsive control over developmental pathways. As demonstrated by recent work in synthetic gene circuits, engineered hormone signalling, and reconfigurable root and shoot traits, the field is now capable of producing highly tailored modifications to plant form and function that were previously unattainable.

Looking ahead, the next major challenge lies in engineering developmental plasticity. As climate change intensifies environmental variability, crops must be able to sense and respond to fluctuating conditions more effectively than many current genotypes allow. Synthetic biology provides a powerful framework for reinstating or augmenting adaptive plasticity using orthogonal circuits that integrate multiple environmental inputs and generate context-appropriate developmental outputs. Mathematical modelling, chassis engineering, and synthetic organelles are poised to further expand this design space, enabling the construction of plants with entirely new regulatory architectures. These innovations will be especially impactful for low-input and smallholder farming systems, where environmental uncertainty is high and adaptive traits can substantially improve resilience.

Realising the full potential of synthetic biology in agriculture will require parallel advances in regulation, public engagement, and translational pipelines. As gene editing and transgenic technologies mature, regulatory frameworks must evolve to focus on traits and risk profiles, rather than the method by which a modification was introduced. Public confidence will also be essential; transparent communication, co-design with growers, and equitable access must remain central priorities. Nonetheless, the accelerating pace of technological development suggests that a new generation of programmable crops—capable of responding dynamically to their environments, producing higher yields under stress, and exhibiting novel architectures—may soon be within reach. In an era of mounting climate and food security challenges, synthetic biology offers one of the most promising and versatile toolkits for building the resilient crops required for the decades to come.

## Data Availability

No new data was generated here.
